# Metabolic Disease Trends With Adhesive Capsulitis in South Texas High-Risk Majority Hispanic Populations

**DOI:** 10.7759/cureus.117682

**Published:** 2026-10-09

**Authors:** Mohammad Khalil, Yolanda Gutierrez, Blake C Martin, Nicholas Bonazza, Robert Schenck, Aamir Ahmad

**Affiliations:** 1 Medicine, The University of Texas Rio Grande Valley School of Medicine, Edinburg, USA; 2 Orthopedic Surgery, University of Arizona College of Medicine, Tucson, USA; 3 Orthopedic Surgery, University of Texas Rio Grande Valley School of Medicine, Edinburg, USA

**Keywords:** adhesive capsulitis, diabetes, frozen shoulder, obesity, rio grande valley

## Abstract

Background

Adhesive capsulitis, or frozen shoulder, is a condition characterized by pain and restricted motion in the shoulder joint. Diabetes is one of the core risk factors for the condition, and obesity has been demonstrated to decrease the age of onset in patients with adhesive capsulitis. This study aims to investigate the prevalence of diabetes and obesity in patients diagnosed with adhesive capsulitis in the Rio Grande Valley, a region with a high prevalence of metabolic disorders.

Methods

A retrospective analysis was conducted on 836 cases of diagnosed adhesive capsulitis. Clinical data regarding shoulder involvement, diabetes status, and obesity measurements were analyzed. Multivariable logistic regression was performed to evaluate independent predictors of bilateral adhesive capsulitis, including diabetes, obesity, age, and sex.

Results

A total of 786 unique patients, with 836 shoulders affected, met inclusion criteria for adhesive capsulitis, with 49 of those patients having bilateral disease. The mean age was 58.92 ± 11.00 years, and 66.4% were female. Ethnicity was recorded for 782 patients, of whom 89.6% (n = 701) identified as Hispanic/Latino. According to BMI measurements, 51.0% of patients were obese, 34.8% were overweight, 13.6% were of healthy weight, and 0.6% were underweight. Diabetes was present in 15.3% (n = 120) of patients. On multivariable analysis, bilateral adhesive capsulitis was significantly more common among diabetic patients, with regression showing an independently associated significant risk (adjusted OR 3.41, 95% CI 1.85-6.30, p < 0.001). Obese patients presented at a younger age than non-obese patients (57.9 vs 60.4 years, p = 0.002).

Conclusion

This study highlights the high prevalence of diabetes and obesity in patients with adhesive capsulitis in the Rio Grande Valley, reinforcing the potential metabolic component in the condition's pathogenesis. Addressing these metabolic risk factors may improve adhesive capsulitis outcomes and overall patient health in similar high-risk populations.

## Introduction

Adhesive capsulitis is a condition characterized by increased inflammation and fibrotic deposition in the glenohumeral joint, resulting in excessive pain and decreased range of motion [1]. This condition severely limits patients' activities of daily living and is accompanied by sleep issues as well due to prolonged pain [2]. Adhesive capsulitis can progress in phases, typically starting with two to nine months of shoulder pain, followed by stiffness for 4-12 months, and lastly the thawing phase, which can last between 5-24 months; however, it is important to note that symptoms can persist for years with chronic pain [3]. The prevalence of this condition ranges between 2-5% and has a higher rate in women (10.1%) compared to men (8.2%), and has a cumulative incidence of 2.4 per 1000 per year [1,2]. 

Interestingly, diabetes mellitus is a significant risk factor for the development of adhesive capsulitis, with studies demonstrating that an increase of 10 mmol/mol of HbA1c increases the odds of adhesive capsulitis by at least 50% [4]. Chronic hyperglycemia has been shown to upregulate inflammatory cytokines such as IL-6 and IL-8, causing inflammation and fibrosis in the glenohumeral joint [5]. Along with diabetes, other comorbidities such as obesity have demonstrated a correlation with adhesive capsulitis. However, there have been varying thoughts on the role obesity plays in the development of adhesive capsulitis. Mendelian studies have found that a genetically predicted BMI increase of 5 kg/m^2 was not associated with adhesive capsulitis [4]. Despite these findings, other studies have demonstrated the prevalence of obesity in adhesive capsulitis in cohorts to be 27% higher compared to the control cohort [6]. It is important to note that most studies available in the literature evaluate obesity and diabetes mellitus in heterogeneous or non-regional cohorts, which limits the applicability to populations that experience high rates of metabolic disease, such as the Rio Grande Valley [7]. 

The Rio Grande Valley has significant rates of metabolic disease, with a diabetes prevalence of 30.7%, which is more than double the national rate of 12.3% [7]. Similarly, in a Cameron County cohort, the obesity rates range between 55-57% having a BMI >30 kg/m^2 [8,9]. With the Rio Grande Valley's unique prevalence of obesity and diabetes, this could likely translate to a substantially elevated burden of adhesive capsulitis in this population. This study aims to evaluate the metabolic characteristics of adhesive capsulitis in patients from the Rio Grande Valley, a region with a high prevalence of metabolic disorders, and assess whether diabetes or obesity influence disease presentation, including bilateral involvement.

A preliminary version of this research was presented as a meeting abstract at the University of Texas Rio Grande Valley School of Medicine Research Symposium in March of 2025. 

## Materials and methods

A retrospective analysis was conducted on the prospectively maintained University of Texas Rio Grande Valley (UTRGV) UTHealth electronic database from January 1, 2014, to April 23, 2025. Inclusion criteria for patients entailed a diagnosis of adhesive capsulitis/frozen shoulder using an ICD-10 code of M75.0. Patients of all ages were included. All patients within the database with an ICD-10 code for adhesive capsulitis during the study period were included, regardless of encounter type. The first encounter associated with an adhesive capsulitis diagnosis was used as the patient's initial adhesive capsulitis encounter. Diagnosis of adhesive capsulitis was based solely on ICD-10 coding. No additional exclusion criteria were applied.

Patients were categorized as having adhesive capsulitis of the right shoulder, left shoulder, or unspecified shoulder. Some patients had bilateral cases of adhesive capsulitis. Laterality was determined based on ICD-10 coding. Patients were counted once as unilateral cases unless both shoulders were affected, in which case they were categorized as having bilateral adhesive capsulitis. Bilateral adhesive capsulitis was defined as involvement of both shoulders and did not require both shoulders to be affected at the same time in order to analyze potential metabolic associations that may be related to patients who may eventually develop bilateral disease. Patients with unspecified shoulder laterality were treated as having unilateral adhesive capsulitis. Included patients with diabetes diagnoses, based on ICD-10 codes, were also noted. Both type 1 and type 2 diabetes mellitus were included based on ICD-10 coding. Diabetes was considered present when the diagnosis was documented at or before the time of adhesive capsulitis diagnosis.

Demographic variables, including age, sex, and ethnicity, were collected. Hispanic/Latino ethnicity was specifically identified. Clinical data regarding diabetes status and obesity measurements were analyzed. Obesity was defined as a BMI greater than 30 kg/m² at the encounter associated with the adhesive capsulitis diagnosis. Continuous variables were summarized as means ± standard deviations and compared using independent samples t-tests. Categorical variables were summarized as frequencies and percentages and compared using chi-square or Fisher's exact tests, as appropriate. Multivariable logistic regression utilizing complete cases (those with a recorded BMI, n = 675) was performed to evaluate independent predictors of bilateral adhesive capsulitis, including diabetes, obesity, age, and sex. Results were reported as odds ratios (ORs) with 95% confidence intervals (CIs). Analyses were conducted using available data, and missing values were not imputed. Patients with missing data for an individual variable were excluded only from analyses involving that specific variable. A two-sided p-value < 0.05 was considered statistically significant.

## Results

A total of 786 unique patients met inclusion criteria for adhesive capsulitis with a mean age at presentation of 58.92 years (SD = 11.00), and 66.4% (n = 522; 95% CI 63.0-69.6) were female. Ethnicity was recorded for 782 patients; among these, 89.6% (n = 701) identified as Hispanic/Latino. 

Shoulder involvement was more frequent in the left shoulder (50.4%, n = 396), followed by the right shoulder (40.8%, n = 321), with 2.5% (n = 20) coded without laterality. Bilateral involvement, defined as documentation of both right and left adhesive capsulitis at any point during the study period, was present in 6.2% (n = 49) of patients. Of these, 4.6% (n = 36) demonstrated bilateral coding within the same encounter. Laterality distribution did not significantly differ by sex (p = 0.074). Due to bilateral involvement, the cases of adhesive capsulitis in our cohort were 836 in a population of 786 patients.

BMI measurements were available for 675 patients (85.9%) who were subsequently analyzed for a multivariable logistic regression. Among patients with documented BMI, 344 (51.0%) were obese, 235 (34.8%) were overweight, 92 (13.6%) were of healthy weight, and four (0.6%) were underweight, as detailed in Table 1. Patients with missing BMI were similar in age (mean 57.53 vs 59.13 years, p=0.21), but were more often male (45.9% vs 31.6%, p=0.004) and diabetic (26/111 (23.4%) vs 94/675 (13.9%), p=0.015). A complete-case approach was used for the multivariable analysis; multiple imputation was not performed. Among 344 obese patients, 22 presented with bilateral cases. Among 331 non-obese patients, 20 were bilateral.

**Table 1:** Demographic and summary data

Category	Quantity
Total cases	836
Shoulder involvement	
Right shoulder	321 (40.8%)
Left shoulder	396 (50.4%)
Both shoulders	49 (6.2%)
Unspecified shoulder	20 (2.5%)
Total patients	786
Demographics	
Female	522 (66.4%)
Male	264 (33.6%)
Mean age	58.9 years
Ethnicity	
Hispanic	701 (89.6%)
Non-Hispanic	81 (10.4%)
Ethnicity not stated	4
Diabetes	
Total patients with diabetes	120 (15.3%)
Bilateral adhesive capsulitis cases in diabetic patients	19
BMI measurements	
Total patients with BMI recorded	675 (85.9%)
Obese patients	344 (51.0%)
Overweight patients	235 (34.8%)
Healthy weight	92 (13.6%)
Underweight patients	4 (0.6%)

Diabetes was documented in 120 patients (15.3%). Bilateral adhesive capsulitis was significantly more common among diabetic patients, occurring in 19 of 120 diabetics (15.8%) compared with 30 of 666 non-diabetic patients (4.5%), corresponding to an unadjusted odds ratio of approximately 4.0 (Fisher's exact p < 0.001). On multivariable logistic regression analysis, diabetes remained independently associated with bilateral adhesive capsulitis (adjusted OR 3.41, 95% CI 1.85-6.30, p < 0.001) (Table 2). Obesity, age, and sex were not independently associated with bilateral disease (Table 3). Patients with diabetes were modestly younger than those without diabetes (56.8 vs 59.3 years, p = 0.022). 

**Table 2:** Bilateral adhesive capsulitis association with diabetic patients

Variable	Diabetes (n = 120)	No diabetes (n = 666)	p-value
Mean age (years)	56.8 ± 10.8	59.3 ± 11.0	0.022
Bilateral adhesive capsulitis	19 (15.8%)	30 (4.5%)	<0.001
Unilateral adhesive capsulitis	101 (84.2%)	636 (95.5%)	-
Odds ratio (bilateral adhesive capsulitis)	3.99 (95% CI 2.16–7.35)	-	-

**Table 3:** Multivariable logistic regression analysis of predictors of bilateral adhesive capsulitis

Variable	Adjusted OR	95% CI	p-value
Diabetes mellitus	3.41	1.85 – 6.30	<0.001
Obesity	0.85	0.48 – 1.52	0.585
Age (per year)	1	0.98 – 1.03	0.71
Female sex	0.75	0.42 – 1.35	0.337

In contrast, obesity was not associated with bilateral involvement among patients with adhesive capsulitis (Fisher's exact p = 1.00), nor did laterality distribution differ significantly by obesity status (χ² p = 0.95). However, obese patients presented at a significantly younger age compared with non-obese patients (57.9 vs 60.4 years, p = 0.002) (Table 4). 

**Table 4:** Association of obesity with age presentation of adhesive capsulitis

Variable	Obese (n = 344)	Non-obese (n = 331)	p-value
Mean age (years)	57.9 ± 10.3	60.4 ± 11.1	0.002

## Discussion

In this study, we found a statistically significant association between bilateral glenohumeral adhesive capsulitis and diabetic patients (15.8%) compared to non-diabetic patients with bilateral glenohumeral adhesive capsulitis (4.5%), with a p-value of p < 0.001. Additionally, we found that patients with diabetes who developed adhesive capsulitis were younger than their non-diabetic counterparts (p=0.022). Our findings reinforce the findings of previous studies which demonstrate an association between adhesive capsulitis and diabetes. Previous studies have reported that patients with diabetes have an increased chance of developing adhesive capsulitis, with some studies suggesting that 30% of adhesive capsulitis patients are also diabetic [4,10]. 

Importantly, our study not only highlights a trend in the Rio Grande Valley between diabetes and the presence of adhesive capsulitis, but it suggests diabetes as a factor that worsens the extent of laterality involvement of disease, as reflected by a significantly higher likelihood of bilateral adhesive capsulitis. This observation suggests that diabetes may influence the severity and distribution of capsular pathology rather than simply acting as a risk factor for disease onset. These findings align with a previous review that concluded adhesive capsulitis patients with diabetes may experience more severe disease courses and worse functional outcomes than non-diabetics [11]. Prior studies suggest that such findings could be due to several biological mechanisms. A hallmark of diabetes, due to the chronic hyperglycemia many patients experience, is the promotion of advanced glycation end products (AGEs); over time, these AGEs could collect in connective tissue, like the shoulder joint capsule, and cause an increase in collagen cross-linkages. Over time, this pathophysiological process would lead to decreased tissue elasticity and capsular stiffness [5,12]. Additionally, the onset of hyperglycemia is often accompanied by chronic inflammation via the increased expression of cytokines such as interleukin-6, interleukin-8, and transforming growth factor-β (TGF-β). Over time, these cytokines would stimulate fibroblast proliferation and extracellular matrix deposition within the capsule, further reducing shoulder joint mobility and increasing pain upon movement [5,13,14]. Previous histopathological studies of adhesive capsulitis patient samples reinforce such mechanisms of disease by displaying increased fibroblast and myofibroblast activity and differentiation within the joint capsule [14,15].

Interestingly, while obesity was also highly prevalent in this cohort (51.0%), there wasn't a strong association between obesity and bilateral adhesive capsulitis in this cohort. Our cohort's obesity rate was highly representative of the population of the Rio Grande Valley, and despite the prevalence, obesity alone did not seem to account for bilateral adhesive capsulitis in patients, highlighting the unique role diabetes might play in this disease pathophysiology. Obese patients in our cohort also presented with adhesive capsulitis at a significantly younger age compared with non-obese patients (57.9 vs 60.4 years), corresponding to a mean difference of 2.5 years (p = 0.002), similar to the findings observed in a large national cohort that reported higher BMIs contributing to higher hazard ratios of adhesive capsulitis in younger populations [6,16]. As mentioned above, in the present study, we also found that patients with diabetes who developed adhesive capsulitis were younger than their non-diabetic counterparts (p=0.022), suggesting an additional factor that may influence the age of presentation for adhesive capsulitis. A possible explanation for such findings may be due to obesity and diabetes causing an earlier onset of symptoms through mechanisms such as systemic inflammation or increased mechanical stress on musculoskeletal structures [13,17]. Other analyses, however, including genetic and Mendelian randomization studies, have failed to establish a causal relationship between BMI and disease development [4,18]. In the Rio Grande Valley, there is an underdiagnosis of diabetes that may also impact the true percentage of diabetic patients who are presenting as obese only [7,9]. On the other hand, metabolic syndrome in the Rio Grande Valley's generally homogeneous population could be associated with unique presentations and symptoms, which could be a higher risk for adhesive capsulitis. Further investigation is required to determine whether obesity in fact does have an impact on the development of adhesive capsulitis as a disease process in the Rio Grande Valley, globally, or whether it's just an indicator of metabolic disease trends in the region. 

Regional context plays an important role when interpreting these findings from the Rio Grande Valley. The Rio Grande Valley is estimated to have one of the largest metabolic disease burdens in the United States, with studies reporting diabetes prevalence around 30% and obesity rates of over 50% in several cohorts [7-9]. In the present study, the prevalence of obesity among patients with adhesive capsulitis matched regional estimates, suggesting that obesity within this cohort largely reflects the background metabolic environment of the region. In contrast, the prevalence of diabetes within this cohort was lower than Rio Grande Valley population estimates, likely due to a few patients who have been diagnosed with diabetes documented outside the institutional EMR. Another contributing factor to the lower-than-anticipated percentage of diabetic patients could be due to the under-diagnosis of diabetes within the region, as highlighted by previous studies [7,9]. Notably, however, diabetes still demonstrated a significant association with bilateral disease, which suggests a more direct and pathophysiologically significant role in adhesive capsulitis severity in the Rio Grande Valley population. These findings highlight the importance of considering regional trends, especially in demographically unique populations when evaluating musculoskeletal disease patterns and suggest that adhesive capsulitis may represent a clinically relevant manifestation of systemic metabolic dysfunction in high-risk populations such as the Rio Grande Valley.

This study has several limitations. First, the retrospective design and reliance on ICD-10 codes may introduce misclassification, as adhesive capsulitis and diabetes diagnoses were not independently confirmed through chart review. Additionally, diagnoses made outside the UTRGV UTHealth system may not have been captured, which could contribute to underestimation of comorbidities such as diabetes. Similarly, the duration and severity of diabetes, as indicated by factors such as date of diagnosis or HbA1C data, were not readily available for most patients, restricting our analysis of how the extent of diabetes is associated with adhesive capsulitis trends. BMI data were also unavailable for a portion of the cohort, limiting obesity-related analyses. Finally, this study represents a predominantly Hispanic/Latino population from the Rio Grande Valley, which may limit the generalizability of these findings to other populations. 

## Conclusions

In summary, diabetes is strongly associated with bilateral adhesive capsulitis, supporting metabolic disease involvement in the extent of adhesive capsulitis. In contrast, obesity appears to influence the timing of presentation rather than disease distribution. In the Rio Grande Valley, where metabolic disease is highly prevalent, these findings underscore the importance of considering diabetes as a key clinical factor in disease severity and management. Further prospective studies are needed to clarify the role of metabolic factors in the development and progression of adhesive capsulitis.

## References

[1] Mullen JP, Hauer TM, Lau EN, Lin A (2025). Adhesive capsulitis of the shoulder. Arthroscopy.

[2] Rangan A, Brealey SD, Keding A (2020). Management of adults with primary frozen shoulder in secondary care (UK FROST): a multicentre, pragmatic, three-arm, superiority randomised clinical trial. Lancet.

[3] Challoumas D, Biddle M, McLean M, Millar NL (2020). Comparison of treatments for frozen shoulder: a systematic review and meta-analysis. JAMA Netw Open.

[4] Green HD, Burden E, Chen J (2024). Hyperglycaemia is a causal risk factor for upper limb pathologies. Int J Epidemiol.

[5] Chen MH, Chen WS (2024). A narrative review of adhesive capsulitis with diabetes. J Clin Med.

[6] Kingston K, Curry EJ, Galvin JW, Li X (2018). Shoulder adhesive capsulitis: epidemiology and predictors of surgery. J Shoulder Elbow Surg.

[7] Millard AV, Graham MA, Mier N (2017). Diabetes screening and prevention in a high-risk, medically isolated border community. Front Public Health.

[8] Fisher-Hoch SP, Rentfro AR, Salinas JJ (2010). Socioeconomic status and prevalence of obesity and diabetes in a Mexican American community, Cameron County, Texas, 2004-2007. Prev Chronic Dis.

[9] Fisher-Hoch SP, Vatcheva KP, Rahbar MH, McCormick JB (2015). Undiagnosed diabetes and pre-diabetes in health disparities. PLoS One.

[10] Zreik NH, Malik RA, Charalambous CP (2016). Adhesive capsulitis of the shoulder and diabetes: a meta-analysis of prevalence. Muscles Ligaments Tendons J.

[11] Dyer BP, Burton C, Rathod-Mistry T, Blagojevic-Bucknall M, van der Windt DA (2021). Diabetes as a prognostic factor in frozen shoulder: a systematic review. Arch Rehabil Res Clin Transl.

[12] Hwang KR, Murrell GA, Millar NL, Bonar F, Lam P, Walton JR (2016). Advanced glycation end products in idiopathic frozen shoulders. J Shoulder Elbow Surg.

[13] Le HV, Lee SJ, Nazarian A, Rodriguez EK (2017). Adhesive capsulitis of the shoulder: review of pathophysiology and current clinical treatments. Shoulder Elbow.

[14] Akbar M, McLean M, Garcia-Melchor E (2019). Fibroblast activation and inflammation in frozen shoulder. PLoS One.

[15] Bunker TD, Anthony PP (1995). The pathology of frozen shoulder. J Bone Joint Surg Br.

[16] Kim JH, Baek JY, Han KD, Kim BS, Kwon HS (2025). Higher body mass index increases the risk of shoulder adhesive capsulitis in young adults: a nationwide cohort study. J Shoulder Elbow Surg.

[17] Qiao Y, Yao X, Zhao Y, Kang Y, Xu C, Zhao J, Zhao S (2023). Increased expression of adipokines in patients with frozen shoulder. Am J Sports Med.

[18] Green HD, Jones A, Evans JP (2021). A genome-wide association study identifies 5 loci associated with frozen shoulder and implicates diabetes as a causal risk factor. PLoS Genet.

